# Psychological contract, psychological capital, career plateau, and job embeddedness among early-career nurses: a serial mediation model

**DOI:** 10.3389/fpsyg.2026.1822548

**Published:** 2026-05-22

**Authors:** Ximei Wang, Yujuan Qin, Qin Mao, Jianying Wang

**Affiliations:** 1 School of Nursing, Henan Medical College, Zhengzhou, China; 2 School of Nursing, Henan Medical University (Xinxiang Medical University), Xinxiang, China

**Keywords:** career plateau, early-career nurses, job embeddedness, psychological capital, psychological contract, serial mediation, suppression effect

## Abstract

**Background:**

Early-career nurses are critical for sustaining the nursing workforce, yet they are particularly vulnerable to stress, role transition difficulties, and turnover. Job embeddedness offers an important lens for understanding why nurses stay, but the psychological mechanisms linking organizational perceptions to embeddedness remain insufficiently understood in this population.

**Methods:**

A multicenter cross-sectional survey was conducted from August to October 2024 in four tertiary hospitals in Henan Province, China. A convenience sample of 337 early-career nurses (≤3 years of clinical experience) completed validated measures of psychological contract, psychological capital, career plateau, and job embeddedness. Serial mediation was tested using the PROCESS macro (Model 6, 5,000 bootstrap samples), controlling for age, marital status, department, number of night shifts per week, and monthly income.

**Results:**

Psychological contract was positively associated with psychological capital and job embeddedness and negatively associated with career plateau (all *p* < 0.01). After adjusting for covariates, psychological contract significantly predicted higher job embeddedness. The indirect effect through psychological capital alone was 0.095 (95% CI: 0.024–0.179), through career plateau alone was 0.193 (95% CI: 0.139–0.249), and the serial indirect effect through psychological capital and career plateau was negative (−0.052, 95% CI: −0.180 to −0.020), indicating a suppression pattern. The total indirect effect was 0.236, and the direct effect remained significant (0.337).

**Conclusions:**

Psychological contract enhances job embeddedness among early-career nurses both directly and through the serial mediating roles of psychological capital and career plateau. Higher psychological capital may, under limited career development resources, heighten perceptions of career plateau and partly suppress the positive impact of psychological contract. Strengthening psychological contract fulfillment while simultaneously providing realistic career development pathways may help retain early-career nurses in tertiary hospitals.

## 1 Introduction

Global shortages in the health workforce have become a persistent concern, with nurse turnover and staffing deficits particularly prominent. Previous studies have reported annual nurse turnover rates ranging from 15.1 to 44.3% (Mafula et al., 2025). The World Health Organization's Global Strategy on Human Resources for Health: Workforce 2030 proposes 4.45 registered nurses per 1,000 population as the minimum benchmark for national health human resources (Seventy-Second World Health Assembly, 2019). Against this backdrop, there remains a gap between the supply and demand of nursing personnel in China (Statistical bulletin on the development of China's health and wellness sector, 2023). Insufficient nurse staffing and frequent turnover not only increase the workload of remaining nurses but may also decrease work engagement, exacerbate burnout, and elevate turnover intention, thereby forming a vicious cycle.

Early-career nurses are typically defined as those who have obtained nursing qualification but have less than 3 years of clinical work experience (Yang et al., 2024). This group represents an important reserve force for the sustainable development of nursing teams (Lili et al., 2021). However, early-career nurses are still undergoing the transition from education to clinical practice and often face high work pressure, limited clinical experience, role ambiguity, and substantial adaptation demands (Jones et al., 2020). Without adequate support, early-career nurses may experience decreased organizational commitment and increased turnover intention (Jinfang et al., 2022; Kim and Yeo, 2021; Liu et al., 2021). Therefore, clarifying the factors associated with early-career nurses' stable connection with their organizations and positions is an important issue in nursing management.

Job embeddedness provides a useful perspective for understanding why employees stay in an organization. It emphasizes the extent to which individuals are connected to their jobs, organizations, and communities through links, fit, and sacrifice (Liu et al., 2022; Mitchell et al., 2001). Existing nursing studies have examined job embeddedness in relation to work environment, leadership, professional identity, job resources, organizational commitment, and turnover-related outcomes (Alnuaimi et al., 2020; Baba et al., 2020; Dechawatanapaisal, 2018; Fan et al., 2024; Goliroshan et al., 2021; Lee and Lee, 2022; Ng et al., 2010). However, less attention has been paid to how early-career nurses' perceptions of organizational promises, psychological resources, and career development constraints operate together in shaping job embeddedness. In particular, the psychological and career-related pathways linking psychological contract fulfillment to job embeddedness among early-career nurses remain insufficiently understood.

In Chinese tertiary hospitals, this issue may be especially salient. Tertiary hospitals usually have relatively hierarchical management structures, formal professional title systems, intensive clinical training requirements, and high workload. Early-career nurses in these settings often face night shifts, rotation, performance evaluation, standardized training, and expectations for long-term professional development at the same time. Whether the organization provides fair treatment, emotional support, training opportunities, and visible career pathways may therefore strongly shape nurses' perceptions of psychological contract fulfillment, career plateau, and job embeddedness.

To address this gap, the present study integrates psychological contract, psychological capital, career plateau, and job embeddedness into a serial mediation framework. Drawing on Social Exchange Theory and Conservation of Resources theory, this study aimed to examine the association between psychological contract and job embeddedness among early-career nurses, and to test the mediating roles of psychological capital and career plateau. By doing so, this study seeks to provide a more nuanced understanding of early-career nurse retention and to offer evidence for targeted nursing management strategies.

## 2 Literature review and hypothesis development

### 2.1 Psychological contract and job embeddedness

Psychological contract refers to an informal but crucial system of exchange relationships and subjective commitments between employees and organizations (Herrera and de Las Heras-Rosas, 2020). In nursing contexts, psychological contract fulfillment reflects nurses' perceptions that the organization has honored implicit or explicit expectations related to fair treatment, work support, career development, recognition, and emotional respect (Cencen et al., 2021; Cioca et al., 2020). When nurses perceive that the organization fulfills these obligations, they may develop stronger trust, organizational identification, and willingness to reciprocate through positive work attitudes and behaviors.

Social Exchange Theory provides a useful explanation for this relationship. According to this theory, employees tend to respond positively when they perceive that the organization provides valued support and fulfills its obligations. In the nursing workforce, previous studies have shown that psychological contract breach or violation is associated with poorer work attitudes and lower job embeddedness, while fulfillment of organizational promises may help retain satisfied and committed nurses (Dechawatanapaisal, 2022; Faraz et al., 2023; He et al., 2023; Herrera and de Las Heras-Rosas, 2020; Jacobs et al., 2020; Rodwell and Ellershaw, 2016; Topa et al., 2022). For early-career nurses, psychological contract fulfillment may be particularly important because they are still forming their expectations of the organization and evaluating whether the hospital is a suitable place for long-term development. When early-career nurses perceive that the organization provides promised support, fair arrangements, and developmental opportunities, they may experience stronger organizational fit, closer work-related links, and greater perceived sacrifice associated with leaving. Accordingly, we proposed:

Hypothesis 1: Psychological contract is positively associated with job embeddedness among early-career nurses.

### 2.2 Psychological capital as a mediator

Psychological capital is a positive and developable psychological state that commonly includes self-efficacy, hope, optimism, and resilience (Broad and Luthans, 2020). It helps individuals cope with stress, maintain motivation, and respond constructively to challenges in the workplace (Huanhuan, 2023; Shuang et al., 2019). For early-career nurses, psychological capital may be especially valuable because they are still adapting to clinical responsibilities, professional norms, interpersonal relationships, and patient care demands.

Psychological contract fulfillment may contribute to the development of psychological capital. When nurses perceive organizational support, fairness, and promise fulfillment, they may feel more confident in their ability to manage clinical work, more hopeful about future growth, and more resilient when encountering difficulties (De Meseguer et al., 2021; Liu et al., 2021). In turn, nurses with higher psychological capital may be more capable of managing role transition, maintaining positive work attitudes, and building stable connections with their units and organizations. From the perspective of Conservation of Resources theory, psychological contract fulfillment can be understood as an organizational resource, while psychological capital represents an individual psychological resource. The accumulation of organizational resources may help individuals build internal resources, which then support job embeddedness. Accordingly, we proposed:

Hypothesis 2: Psychological capital mediates the association between psychological contract and job embeddedness among early-career nurses.

### 2.3 Career plateau as a mediator

Career plateau generally refers to employees' perceptions that opportunities for promotion, professional growth, or skill development have become limited at a certain stage of their careers (Ference et al., 1977). Previous organizational studies have shown that career plateau is associated with lower job satisfaction, reduced organizational commitment, and higher turnover intention (DiRenzo et al., 2022; Farghaly Abdelaliem and Ibrahim El-Sayed, 2022; Kim and Kim, 2021; Hofstetter and Cohen, 2014). In nursing, career plateau is particularly relevant because clinical nurses' career development is often closely tied to hierarchical promotion systems, professional title evaluation, specialty training opportunities, rotation arrangements, continuing education, and access to professional development resources.

Nursing-specific studies further suggest that career plateau may have important implications for nurse retention. For example, studies on nurses' career plateau have shown that limited career development opportunities, insufficient organizational support, unclear growth pathways, and repetitive clinical work may contribute to disappointment, uncertainty about the future, reduced job satisfaction, and resignation intention (Zhu et al., 2021; Zhu and Li, 2023). Other studies have also linked nurses' career plateau with job embeddedness, self-efficacy, organizational commitment, and turnover intention (Abd-Elrhaman et al., 2020; Kim and Kang, 2015). These findings indicate that career plateau is not only a general organizational phenomenon, but also a practical problem in nursing management.

For early-career nurses, career plateau may appear earlier than expected if they perceive that the organization cannot provide promised training, fair development opportunities, or visible growth pathways. This perception may weaken their sense of organizational fit, reduce their emotional attachment to the workplace, and decrease the perceived sacrifice associated with leaving. Psychological contract fulfillment may reduce career plateau by providing nurses with clearer expectations, developmental support, and fairer organizational arrangements. Conversely, when developmental promises are not fulfilled, nurses may be more likely to perceive career stagnation. Accordingly, we proposed:

Hypothesis 3: Career plateau mediates the association between psychological contract and job embeddedness among early-career nurses.

### 2.4 Serial mediating roles of psychological capital and career plateau

Psychological capital and career plateau may not operate independently. Conservation of Resources theory suggests that individuals strive to obtain, retain, and protect valuable resources, and that stress may occur when valued resources are insufficient, threatened, or lost (Hobfoll et al., 2018). From this perspective, psychological contract fulfillment may provide early-career nurses with organizational support, fair exchange, and developmental resources, thereby promoting psychological capital. Psychological capital may then help nurses manage role transition, work pressure, and uncertainty, which may further support job embeddedness.

However, the relationship between psychological capital and career plateau may be more complex in the early career stage. Early-career nurses with higher psychological capital may have stronger motivation, higher achievement expectations, and clearer aspirations for professional growth. When organizational career resources, promotion opportunities, or skill-development pathways are sufficient, psychological capital may help nurses overcome difficulties and reduce perceptions of stagnation. However, when these organizational resources are limited, nurses with higher psychological capital may become more sensitive to the discrepancy between their career expectations and organizational reality. In this situation, psychological capital may not necessarily reduce career plateau; instead, it may heighten the perception of career stagnation under constrained career development conditions.

This possibility provides a theoretical basis for expecting that the serial pathway involving psychological capital and career plateau may produce complex or even suppressing effects. In other words, psychological contract fulfillment may enhance job embeddedness through psychological capital, but the positive role of psychological capital may be partially offset if higher psychological resources are accompanied by stronger perceptions of unmet career development expectations. Accordingly, we proposed:

Hypothesis 4: Psychological capital and career plateau play serial mediating roles in the association between psychological contract and job embeddedness among early-career nurses.

The direction and magnitude of this serial pathway may vary depending on the match between individual psychological resources and organizational career development resources.

## 3 Methods

### 3.1 Study design and setting

This multicenter cross-sectional questionnaire survey was conducted from August to October 2024 in Henan Province, China. One provincial tertiary hospital from each of four regions (east, west, central and north; there is no provincial tertiary hospital in the south) was included based on feasibility and willingness to participate, resulting in four participating hospitals. Within each hospital, the nursing administration department assisted with on-site recruitment and data collection. The study followed the STROBE reporting guideline for cross-sectional observational studies. Because all variables were measured at a single time point, the cross-sectional design does not allow conclusions about temporal ordering or causality. Therefore, the mediation model in this study should be interpreted as a theory-driven statistical model of associations rather than evidence of causal mediation.

### 3.2 Participants and sampling

The sample size was first estimated based on the prevalence formula for cross-sectional studies. A previous study reported a turnover rate of 12.7% among early-career nurses (Lu et al., 2020). Using a two-sided α of 0.05, *p* = 0.127, an allowable error of 0.05, and a design effect of 1, the minimum sample size was calculated as 170 according to the formula: *n* = *Z*^2^α/2 × *p* × (1 – *p*) × DEFF / *d*^2^.

After considering a potential non-response or invalid response rate of 20%, the target sample size was no fewer than 213. Because the main analysis of this study involved serial mediation, we further considered the adequacy of the final sample for regression-based mediation analysis. The final sample of 337 participants exceeded the minimum sample size estimated for the cross-sectional survey and provided an acceptable basis for multiple regression and bootstrap-based indirect effect testing. Nevertheless, because the original sample size estimation was not based on a mediation-specific power analysis, future studies should use Monte Carlo simulation or other mediation-specific methods to estimate the required sample size for indirect effects more precisely.

### 3.3 Measures

#### 3.3.1 Demographic characteristics

A self-designed demographic questionnaire was developed based on the research aims and target population. It collected information on gender, age, marital status, number of children, highest education, department, number of night shifts per week, frequency of overtime on days off, monthly income, preference for the current department, and type of employment.

#### 3.3.2 Psychological contract

Psychological contract was measured using the scale developed by Hui et al. (2004), which assesses the fulfillment of psychological contract across three dimensions (balanced, relational, and transactional). The original instrument was used to evaluate Chinese employees' perceptions of psychological contract fulfillment. In this study, cross-cultural adaptation was conducted. The original English items were translated and then back-translated by professionals to preserve their meaning, followed by expert review and revision by specialists in nursing and psychology. The final version included 26 items across 3 dimensions. A small pilot study showed satisfactory reliability and validity. The scale uses a 6-point Likert response format (1 = “strongly disagree” to 6 = “strongly agree”), with total scores ranging from 26 to 156; higher scores indicate a higher level of perceived psychological contract fulfillment. Cronbach's alpha coefficients for the total scale and its dimensions ranged from 0.953 to 0.958 in the present study.

#### 3.3.3 Job embeddedness

Job embeddedness was assessed using the Nurse Job Embeddedness Scale originally developed by Mitchell et al. (2001) and adapted to the Chinese context by Qian (2017). The instrument comprises 20 items across four dimensions: organizational fit, organizational affect, community harmony, and career sacrifice. A 5-point Likert response format is used (1 = “strongly disagree” to 5 = “strongly agree”), yielding a total score range of 20–100; higher scores reflect higher levels of job embeddedness. The original Chinese version demonstrated good reliability and validity, with a Cronbach's alpha of 0.919. In the present sample, Cronbach's alpha for the total scale was 0.971.

#### 3.3.4 Psychological capital

Psychological capital was measured using the Psychological Capital Questionnaire developed by Luthans et al. (2004) and adapted for Chinese nurses by Hong and Zhonghua (2010). The scale includes 20 items across four dimensions: self-efficacy (items 1–6), hope (items 7–12), resilience (items 13–17), and optimism (items 18–20). A 6-point Likert response format is used (1 = “strongly disagree” to 6 = “strongly agree”), with total scores ranging from 20 to 120. All items are positively worded; higher scores indicate higher levels of psychological capital. Cronbach's alpha coefficients for the total scale and its dimensions ranged from 0.707 to 0.927 in previous research. In the present study, Cronbach's alpha for the total scale was 0.986.

#### 3.3.5 Career plateau

Career plateau was assessed using the scale originally developed by Millian (1992) and revised by Man (2012) based on domestic and international theories on career plateau and the Chinese cultural context. On the basis of the original 12 items, 14 items were added, yielding a total of 26 items across four dimensions: structural plateau (9 items), attitudinal plateau (7 items), skill plateau (5 items), and content plateau (5 items). A 6-point Likert response format is used (1 = “strongly disagree” to 6 = “strongly agree”), with higher scores indicating higher levels of perceived career plateau. The revised Chinese scale showed good internal consistency, with Cronbach's alpha of 0.821. In the present study, Cronbach's alpha for the total scale was 0.883.

### 3.4 Data collection and quality control

Prior to data collection, approval and support were obtained from the relevant departments at each participating hospital to ensure smooth questionnaire distribution. Investigators received standardized training on the study purpose, procedures, and ethical requirements. Unified instructions were provided, and any questions raised by participants were clarified. Informed consent was obtained, and participants were assured of voluntary participation and confidentiality.

Paper-based questionnaires were distributed on-site and collected immediately after completion. Questionnaire items were set as mandatory to reduce missing data. Completed questionnaires were collected on-site by designated personnel. Two researchers independently checked the questionnaires to identify invalid responses (e.g., excessively short completion time, highly patterned responses or logical inconsistencies), which were excluded to ensure data quality. Data entry and analysis were conducted under the guidance of statisticians.

### 3.5 Statistical analysis

Data were entered into Excel and analyzed using SPSS 27.0. Descriptive statistics were first used to summarize demographic characteristics and the main study variables. Continuous variables were presented as mean ± standard deviation or median and interquartile range, depending on distributional characteristics. Independent-samples t tests, one-way ANOVA, or non-parametric tests were used to compare job embeddedness across demographic groups where appropriate.

Because all focal variables were measured using self-report questionnaires, common method bias was assessed using Harman's single-factor test. To further examine the measurement structure and discriminant validity of the main constructs, confirmatory factor analyses were conducted. A hypothesized four-factor model was compared with alternative models, and a common latent factor model was estimated to evaluate potential common method variance. HTMT values were also examined to assess discriminant validity.

Pearson correlation analysis was then used to examine the bivariate associations among psychological contract, psychological capital, career plateau, and job embeddedness. Multiple linear regression analyses were conducted to examine the associations among the main variables while controlling for age, marital status, department, number of night shifts per week, and monthly income. Multicollinearity was assessed using variance inflation factors.

Finally, the PROCESS macro for SPSS, Model 6 (Hayes, 2015), was used to examine whether the observed associations among psychological contract, psychological capital, career plateau, and job embeddedness were consistent with the proposed serial mediation framework. Bootstrap sampling with 5,000 repetitions and 95% confidence intervals was used to evaluate the statistical indirect pathways involving psychological capital and career plateau. Given the cross-sectional nature of the data, the estimated indirect effects were interpreted as theory-driven statistical indirect pathways rather than causal effects. A two-tailed *p* value < 0.05 was considered statistically significant.

### 3.6 Ethics approval and consent to participate

This study was conducted in accordance with the ethical principles of the Declaration of Helsinki. Ethical approval was obtained from the Ethics Committee of Xinxiang Medical University, Henan Province, China (approval No. XXLL-20240380). Permission for data collection was also obtained from the nursing administration departments of all participating hospitals. Before questionnaire administration, the investigators explained the purpose, procedures, potential risks and benefits of the study to eligible early-career nurses and emphasized that participation was entirely voluntary. Written informed consent was obtained from all participants. They had the right to refuse participation or withdraw from the study at any time without any negative consequences.

## 4 Results

### 4.1 Common method bias

To examine potential common method bias, Harman's single-factor test was conducted using unrotated exploratory factor analysis. Ten factors with eigenvalues greater than 1 were extracted, and the first factor accounted for 26.884% of the total variance, which is below the commonly used 40% threshold. This suggests that common method variance is unlikely to be the dominant source of the observed associations. However, given the cross-sectional self-report design, residual method bias and substantial shared variance across constructs cannot be entirely ruled out. Therefore, confirmatory factor analyses (CFA) and a common latent factor (CLF) model were further conducted to rigorously evaluate the measurement structure.

### 4.2 Measurement model and discriminant validity (CFA)

Because the scales used in this study contained a relatively large number of items, item parceling was applied to improve estimation stability and reduce single-item measurement error. Three parcels were created for each latent construct (psychological contract, psychological capital, career plateau, and job embeddedness), and CFAs were performed using maximum likelihood estimation.

The hypothesized four-factor model demonstrated better fit than competing models [χ^2^(48) = 390.651, CFI = 0.952, TLI = 0.934, RMSEA = 0.146]. In contrast, the one-factor model showed poor fit [χ^2^(54) = 1531.732, CFI = 0.794, TLI = 0.748, RMSEA = 0.285]. The improvement from the one-factor to the four-factor model was statistically significant (Δχ^2^ = 1,141.081, Δdf = 6, *p* < 0.001), supporting the distinctiveness of the four focal constructs.

To further address potential common method variance, a CLF model was estimated by adding a common latent factor loading on all parcels within the four-factor structure. Model fit improved substantially [χ^2^(36) = 163.893, CFI = 0.982, TLI = 0.967, RMSEA = 0.103], indicating that shared method variance contributed to the covariance structure but did not eliminate the four-factor solution. In addition, item-level HTMT indices showed relatively high values for several construct pairs (e.g., psychological contract–psychological capital, psychological contract–job embeddedness, and career plateau–job embeddedness, all > 0.90), suggesting substantial shared variance between some constructs. Taken together, the CFA, competing models, and CLF results support the four-factor measurement structure while also indicating that residual method bias and construct overlap should be considered when interpreting the findings. Although the four-factor model fitted the data better than the one-factor model, the absolute fit indices should be interpreted cautiously. In particular, the RMSEA values of the four-factor model and the CLF model remained above commonly recommended cut-off values, suggesting that the measurement model did not achieve ideal absolute fit. In addition, several HTMT values exceeded 0.90, indicating substantial shared variance between some constructs. These findings suggest that psychological contract, psychological capital, career plateau, and job embeddedness were empirically distinguishable to some extent, but not clearly separated in all respects. Therefore, subsequent structural and mediation analyses should be interpreted with caution, especially regarding potential construct overlap and residual common method variance.

### 4.3 Participant characteristics and job embeddedness

Univariate analyses showed that job embeddedness scores differed significantly by age, marital status, department, number of night shifts per week, and monthly income (*p* < 0.05). Detailed results are presented in Table 1.

**Table 1:** Participant characteristics and univariate analysis of job embeddedness (*n*= 337).

Variable	Category	*n*	Job embeddedness (M ±SD/M[P_25_, P_75_])	t/F/H	*p*
Sex	Male	47	4.060 ± 0.79	−1.212^a^	0.226
	Female	290	4.200 ± 0.72		
Age (years)	< 22	72	3.790 ± 0.85	9.512^b^	< 0.001
	22	153	4.280 ± 0.69		
	25	89	4.290 ± 0.65		
	>30	23	4.310 ± 0.51		
Marital status	Unmarried	195	4.20 (4.0,4.7)	8.574^c^	0.014
	Married	142	4.35 (4.0,4.8)		
Number of children	None	219	4.20 (4.0,4.7)	4.668^c^	0.097
	One child	54	4.30 (4.0,4.8)		
	Two or more	64	4.43 (4.1,4.7)		
Highest education	Secondary technical school	1	4.10 (4.1,4.1)	3.389^c^	0.335
	Junior college	16	4.53 (4.1,5.1)		
	Bachelor's degree	308	4.30 (4.0,4.7)		
	Master's degree or above	12	4.43 (4.0,4.8)		
Department	Internal medicine	106	4.280 ± 0.61	3.230^b^	0.001
	Surgery	61	4.070 ± 0.90		
	Gynecology	12	4.380 ± 0.35		
	Pediatrics	7	4.510 ± 0.42		
	Intensive care unit	57	4.280 ± 0.69		
	Operating room	29	3.610 ± 0.89		
	Emergency department	39	4.200 ± 0.65		
	Infectious diseases	12	4.200 ± 0.27		
	Others	14	4.220 ± 0.89		
Number of night shifts(week)	None	74	4.290 ± 0.64	3.457^b^	0.033
	1–2 times	124	4.130 ± 0.75		
	>2 times	139	4.040 ± 0.83		
Overtime on days off	None	109	4.270 ± 0.66	1.038^b^	0.376
	1–2 times	161	4.230 ± 0.80		
	3–4 times	36	4.130 ± 0.75		
	≥5 times	31	4.100 ± 0.80		
Monthly income (CNY)	< 4000	84	3.970 ± 0.82	5.572^b^	0.004
	4000~	194	4.210 ± 0.72		
	≥8000	59	4.360 ± 0.57		
Type of employment	Contract staff	286	4.170 ± 0.73	1.058^b^	0.367
	Agency/dispatched staff	9	3.980 ± 0.98		
	Permanent staff (establishment)	32	4.360 ± 0.59		
	Others	10	4.030 ± 0.80		

a: *t*-test; b: one-way ANOVA; c: Kruskal–Wallis H test.

### 4.4 Descriptive statistics and correlations among main variables

Pearson correlation analysis indicated significant associations among all pairs of variables. Psychological contract was positively correlated with psychological capital (*r* = 0.918, *p* < 0.01) and job embeddedness (*r* = 0.901, *p* < 0.01), and negatively correlated with career plateau (*r* = −0.753, *p* < 0.01). Career plateau was negatively associated with psychological capital (*r* = −0.648, *p* < 0.01) and job embeddedness (*r* = −0.803, *p* < 0.01). Job embeddedness was positively correlated with psychological capital (*r* = 0.840, *p* < 0.01). Descriptive statistics and correlations are presented in Table 2.

**Table 2:** Descriptive statistics and Pearson correlations among main variables (*n*= 337, *r*).

Variable	Mean ±SD	1	2	3	4
1 Psychological contract	4.6611 ± 0.133	1			
2 Psychological capital	4.5431 ± 0.211	0.918^**^	1		
3 Career plateau	3.1880 ± 0.540	−0.753^**^	−0.648^**^	1	
4 Job embeddedness	4.1780 ± 0.730	0.901^**^	0.840^**^	−0.803^**^	1

^**^p < 0.01.

The correlations among several focal constructs were notably high. In particular, psychological contract was strongly correlated with psychological capital and job embeddedness, suggesting that early-career nurses who perceived higher fulfillment of organizational obligations also tended to report stronger positive psychological resources and closer attachment to their organization. While these associations are theoretically meaningful, their magnitude also raises methodological and conceptual concerns. High correlations may partly reflect shared method variance due to the use of self-report questionnaires, a general positive evaluation tendency, or overlapping construct content. Therefore, we further conducted CFA-based competing model comparisons, a CLF model, HTMT analysis, and multicollinearity diagnostics. These additional analyses were used to evaluate whether the focal variables could be treated as empirically distinguishable constructs and to guide cautious interpretation of the subsequent regression and mediation results.

### 4.5 Regression analyses

Multiple linear regression analyses were performed to explore the potential roles of psychological capital and career plateau in the relationship between psychological contract and job embeddedness. Psychological capital, career plateau, and job embeddedness were entered as dependent variables in separate models. Age, marital status, department, number of night shifts per week, and monthly income were included as covariates in all models. All variance inflation factors were below 10, indicating that multicollinearity was not severe.

As shown in Table 3, psychological contract positively predicted psychological capital after controlling for covariates (*B* = 0.974, *p* < 0.01, *R*^2^ = 0.847). Psychological contract negatively predicted career plateau (*B* = −0.478, *p* < 0.01), whereas psychological capital positively predicted career plateau (*B* = 0.133, *p* < 0.01; *R*^2^ = 0.599). When job embeddedness was used as the dependent variable, psychological contract remained a significant positive predictor in the first step after adjusting for covariates (β = 0.523, *p* < 0.01, *R*^2^ = 0.816). In the second step, after simultaneously entering psychological capital and career plateau, psychological contract (β ≈ 0.299, *p* < 0.01) and psychological capital (β = 0.162, *p* < 0.01) continued to show significant positive associations with job embeddedness, whereas career plateau showed a significant negative association (β ≈ −0.926, *p* < 0.01). The model's explanatory power was further enhanced (*R*^2^ = 0.853).

**Table 3:** Multiple regression analyses of psychological contract, psychological capital, career plateau, and job embeddedness (*n*= 337).

Predictor	PC			CP			JE (Model 1)			JE (Model 2)		
	SE	B	*t*	SE	B	*t*	SE	B	*t*	SE	B	*t*
Age	0.047	0.072	1.536	0.034	−0.060	−1.751	0.031	0.011	0.344	0.028	−0.017	−0.591
Marital status	0.074	−0.009	−0.122	0.054	−0.021	−0.391	0.049	0.027	0.561	0.044	0.019	0.441
Department	0.010	−0.004	−0.429	0.007	−0.013	−1.826	0.006	0.005	0.817	0.006	0.000	0.043
Night shifts/week	0.034	−0.063	−1.845	0.025	−0.036	−1.463	0.023	0.031	1.369	0.020	0.019	0.930
Monthly income	0.046	0.026	0.573	0.033	−0.019	−0.562	0.030	0.038	1.264	0.027	0.029	1.087
PC	0.024	0.974	40.895	0.043	−0.478	−11.246	0.016	0.573	36.380	0.041	0.337	8.231
PsyCap				0.040	0.133	3.340				0.033	0.098	2.944
CP										0.045	−0.403	−8.926
*R* ^2^	0.847			0.599			0.816			0.853		
Adjusted *R*^2^	0.845			0.590			0.813			0.849		
F	305.453^***^			70.120^***^			244.710^***^			237.931^***^		

PC, psychological contract; PsyCap, psychological capital; CP, career plateau; JE, job embeddedness; B, unstandardized coefficient; SE, standard error. All models controlled for age, marital status, department, number of night shifts per week, and monthly income. Variance inflation factors were below 10 in all models. ^***^p < 0.001.

To further clarify the independence of these associations, partial correlations were calculated. After controlling for psychological contract, the correlation between psychological capital and career plateau changed from a negative zero-order correlation to a positive partial correlation (*r* = 0.165, *p* < 0.01), consistent with the regression finding that psychological capital positively predicted career plateau. This pattern suggests the presence of a suppression effect and provides a statistical basis for the subsequent analysis of serial mediation.

### 4.6 Serial mediation analysis

**Figure 1:**
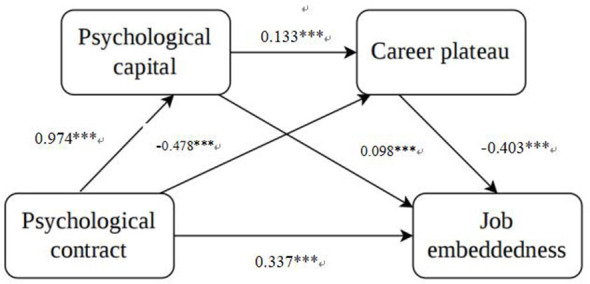
Serial mediation model linking psychological contract, psychological capital, career plateau, and job embeddedness. Unstandardized path coefficients are shown. Covariates included age, marital status, department, number of night shifts per week, and monthly income. ***Indicates statistical significance at *p* < 0.001.

The statistical indirect pathways involving psychological capital and career plateau were examined using the PROCESS macro Model 6 with 5,000 bootstrap samples and 95% confidence intervals, controlling for age, marital status, department, number of night shifts per week, and monthly income. Because the data were cross-sectional, the results are presented as theory-driven indirect associations rather than causal mediation effects. The results are shown in Figure 1 and Table 4.

**Table 4:** Indirect, direct, and total effects in the serial mediation model (*n*= 337).

Mediation pathway	Effect	BootSE	Bootstrap 95% CI	
			Lower	Upper
Ind1	0.095	0.039	0.024	0.179
Ind2	0.193	0.028	0.139	0.249
Ind3	−0.052	0.016	−0.180	−0.020
Direct effect	0.337	0.041	0.257	0.418
Total indirect effect	0.236	0.048	0.145	0.334
Total effect	0.573	0.016	0.542	0.604

Ind1 = PC → PsyCap → JE; Ind2 = PC → CP → JE; Ind3 = PC → PsyCap → CP → JE. PC, psychological contract; PsyCap, psychological capital; CP, career plateau; JE, job embeddedness; Boot SE, bootstrap standard error; CI, confidence interval.

The total effect of psychological contract on job embeddedness was significant (95% CI: 0.542–0.604, *p* < 0.01). When psychological capital and career plateau were included as mediators, the direct effect of psychological contract on job embeddedness remained significant (95% CI: 0.257–0.418, *p* < 0.01), indicating that psychological contract exerted an independent positive effect on job embeddedness beyond the mediating variables. The total indirect effect was 0.236 (95% CI: 0.145–0.334), suggesting that psychological capital and career plateau jointly played important mediating roles.

Regarding specific pathways, the indirect effect through psychological capital alone (psychological contract → psychological capital → job embeddedness) was 0.095 (95% CI: 0.024–0.179), indicating a significant mediating role of psychological capital. The indirect effect through career plateau alone (psychological contract → career plateau → job embeddedness) was 0.193 (95% CI: 0.139–0.249), indicating a significant mediating role of career plateau. The serial indirect pathway through psychological capital and career plateau (psychological contract → psychological capital → career plateau → job embeddedness) was negative and significant (effect = −0.052, 95% CI: −0.180 – −0.020). This result is consistent with the suppression pattern observed in the partial correlation analyses and suggests that, among early-career nurses, psychological capital may, under certain conditions, influence career plateau in ways that modify both the direction and magnitude of the effect of psychological contract on job embeddedness.

## 5 Discussion

### 5.1 Main findings and theoretical interpretation

This multicenter cross-sectional study confirmed that psychological contract had a significant positive effect on job embeddedness among early-career nurses and influenced job embeddedness through the serial mediating roles of psychological capital and career plateau. Psychological contract directly enhanced job embeddedness and indirectly promoted job embeddedness by increasing psychological capital and reducing the negative impact of career plateau. An unexpected finding was that nurses with higher psychological capital, under the same level of psychological contract, were more likely to experience career plateau, suggesting a suppression effect.

This study examined the association between psychological contract and job embeddedness among early-career nurses and explored the statistical indirect pathways involving psychological capital and career plateau. The findings showed that psychological contract was positively associated with job embeddedness, and that psychological capital and career plateau were statistically linked to this association. These results are broadly consistent with previous studies showing that psychological contract fulfillment or breach is closely related to nurses' job embeddedness and work attitudes (He et al., 2023; Li et al., 2022; Rodwell and Johnson, 2022).

From the perspective of Social Exchange Theory, when early-career nurses perceive that the organization fulfills its promises and provides fair treatment, support, and development opportunities, they may be more likely to respond with positive work attitudes and stronger organizational attachment (Ahmad et al., 2022). For nurses in the early stage of their careers, psychological contract fulfillment may be especially important because they are still forming expectations about the organization and evaluating whether the hospital is a suitable place for long-term development. A fulfilled psychological contract may therefore strengthen organizational fit, work-related links, and the perceived sacrifice associated with leaving, thereby supporting job embeddedness (Dechawatanapaisal, 2018; Siyu et al., 2024).

The mediating pathway through psychological capital is also theoretically meaningful. Psychological capital reflects a positive and developable psychological resource, including self-efficacy, hope, optimism, and resilience. Previous studies have shown that psychological capital is associated with lower turnover intention and more positive work attitudes among nurses (Flinkman et al., 2023; Wang et al., 2024; Xiao et al., 2022). In the present study, psychological contract fulfillment may be understood as an organizational resource that helps early-career nurses build individual psychological resources. This interpretation is consistent with Conservation of Resources theory, which suggests that the acquisition of valuable resources may promote further resource gains (Huahua et al., 2022). It is also consistent with the broaden-and-build theory, which proposes that positive psychological states can broaden individuals' thought–action repertoires and support adaptive coping (Fredrickson, 2001). For early-career nurses, sufficient psychological capital may help them adapt to clinical work, regulate negative emotions, and reduce burnout during the role transition period (Yao et al., 2022).

However, because this study used a cross-sectional self-report design, these findings should be interpreted as theory-driven statistical associations rather than evidence of causal processes.

### 5.2 The role of career plateau and the suppression pattern

Career plateau appeared to be an important pathway linking psychological contract and job embeddedness. Previous studies have shown that career plateau is associated with lower job satisfaction, reduced organizational commitment, weaker work engagement, and higher turnover intention (Farghaly Abdelaliem and Ibrahim El-Sayed, 2022; Polat et al., 2025; Zhu et al., 2021). In the nursing context, this issue may be particularly important because nurses' career development is often closely related to professional title evaluation, specialty training, continuing education, promotion opportunities, and clinical role expansion. According to the career development stages of nurses, early-career nurses are generally in the stages of scanning the environment and assessing reality and self (Donner and Wheeler, 2001). At this stage, they are still constructing professional identity and evaluating whether their organization can support their long-term growth.

For this reason, career plateau may have a direct and immediate relevance to early-career nurses' job embeddedness. If early-career nurses perceive limited development opportunities, unclear promotion criteria, repetitive clinical tasks, or insufficient professional recognition, they may experience a mismatch between career expectations and organizational reality. This perception may weaken organizational fit and reduce the perceived sacrifice associated with leaving. Therefore, compared with psychological capital, the indirect pathway through career plateau may be stronger because it more directly reflects nurses' judgments about organizational career opportunities and future professional growth.

The negative serial indirect pathway through psychological capital and career plateau suggests a possible suppression pattern (Zhonglin et al., 2022). Previous research has often suggested that psychological capital is associated with lower career plateau (Chang et al., 2024). However, our findings indicate that the relationship may be more complex among early-career nurses. One possible explanation is that nurses with higher psychological capital may have stronger achievement motivation and clearer expectations for professional growth. When organizational career resources are sufficient, psychological capital may help nurses overcome challenges and reduce perceptions of stagnation. However, when promotion opportunities, training resources, or skill-development pathways are limited, higher psychological capital may make nurses more aware of the gap between their career expectations and organizational reality. In this situation, psychological capital may become a “double-edged sword,” partly offsetting the positive association between psychological contract and job embeddedness through heightened perceptions of career plateau (Jin et al., 2022).

This finding suggests that nurse retention strategies should not rely solely on individual-level psychological interventions. Although psychological capital is valuable for helping early-career nurses cope with stress, it may not fully compensate for limited organizational career resources. Nurse managers should therefore address both individual psychological resources and structural career development barriers when seeking to improve job embeddedness.

Another noteworthy finding is that the indirect pathway through career plateau appeared stronger than the pathway through psychological capital. This may be because career plateau is more directly related to nurses' judgments about organizational career opportunities, promotion prospects, and future professional growth. For early-career nurses, decisions about whether to remain embedded in the organization may depend not only on their internal psychological resources, but also on whether the organization provides visible and attainable development pathways. Psychological capital may help nurses cope with work stress and maintain positive attitudes, but its positive role may be constrained when organizational career resources are insufficient. In contrast, career plateau directly reflects the perceived mismatch between career expectations and organizational opportunities, which may have a more immediate association with job embeddedness. This finding suggests that nurse retention strategies should not rely solely on individual-level psychological interventions, but should also address structural career development barriers.

### 5.3 Interpretation of construct overlap and discriminant validity

An important issue that should be considered when interpreting these findings is the discriminant validity of the focal constructs. Psychological contract, psychological capital, and job embeddedness were highly correlated, and several HTMT values exceeded the recommended threshold of 0.90. Although the CFA results showed that the four-factor model performed better than the one-factor model, the RMSEA values indicated that the absolute model fit was not ideal. These results suggest that the constructs in this study may share a considerable amount of variance, possibly because early-career nurses' perceptions of organizational support, positive psychological resources, and attachment to the workplace are closely intertwined in practice. Therefore, the mediation model should not be interpreted as definitive evidence of fully independent psychological processes. Rather, it should be understood as a theory-driven statistical representation of closely related psychological and organizational perceptions.

### 5.4 Implications for nursing management

The findings have several implications for nursing management in Chinese tertiary hospitals. First, nurse managers should strengthen psychological contract fulfillment during the first 3 years of employment. This can be achieved through regular expectation communication, transparent explanation of ward rotation and night-shift arrangements, timely feedback after performance evaluation, and visible delivery of promised training and support. For early-career nurses, unmet expectations may accumulate quickly during the role transition period; therefore, head nurses and nursing administrators should identify expectation gaps early and respond before they develop into perceived psychological contract breach.

Second, psychological capital interventions should be combined with concrete career development resources. Resilience training, peer support, and mentoring may help early-career nurses cope with stress, but these interventions may be insufficient if nurses cannot see realistic development opportunities. Hospitals should provide staged career development interviews, mentor-based growth plans, competency portfolios, and clear information about professional title evaluation, specialty nurse training, continuing education, and promotion pathways.

Third, career plateau should be monitored as an early warning signal for reduced job embeddedness. In Chinese tertiary hospitals, early-career nurses may experience career stagnation not only because of limited promotion opportunities, but also because of repetitive clinical tasks, unclear specialty development, and insufficient autonomy. Nurse managers may reduce early perceptions of plateau by offering diversified role experiences, cross-unit learning opportunities, participation in quality improvement projects, and individualized career planning. These measures may help align nurses' psychological resources with organizational development opportunities and reduce the expectation–reality gap.

### 5.5 Limitations and future research

This study has several limitations. First, the cross-sectional design precludes causal inferences regarding the relationships among variables. Future research could employ longitudinal or experimental designs to further clarify causality. Second, participants were recruited using multicenter convenience sampling from provincial tertiary hospitals in a single province, which may introduce selection bias and limit the generalizability of the findings to nurses in other regions or different levels of healthcare institutions.

Third, all variables were measured using self-report questionnaires at a single time point, which may have introduced common method variance. Although Harman's single-factor test, CFA competing models, and the CLF model were conducted, these procedures cannot fully eliminate the influence of shared method variance. More importantly, the high correlations among several focal constructs and HTMT values above 0.90 indicate possible construct overlap and limited discriminant validity. Therefore, the estimated indirect pathways may partly reflect shared evaluative tendencies or overlapping construct content rather than completely distinct psychological mechanisms. Future studies should use multi-source data, time-lagged designs, and more refined measurement tools to further examine the distinctiveness of these constructs.

## 6 Conclusions

This study aimed to examine the association between psychological contract and job embeddedness among early-career nurses and to explore the statistical indirect pathways involving psychological capital and career plateau. The findings showed that psychological contract was positively associated with job embeddedness, and that psychological capital and career plateau were statistically linked to this association. Career plateau appeared to be a particularly important pathway, and the negative serial indirect pathway suggested a possible suppression pattern.

These findings indicate that early-career nurses' job embeddedness may be closely related not only to whether organizational promises are fulfilled, but also to whether their psychological resources are matched by realistic career development opportunities. For nursing managers in tertiary hospitals, efforts to cultivate psychological capital should be accompanied by transparent promotion criteria, accessible training resources, and individualized career planning in order to reduce perceptions of career plateau.

However, the findings should be interpreted cautiously because of the cross-sectional self-report design, high correlations among several constructs, and limitations in discriminant validity. Future studies should use longitudinal or time-lagged designs to establish temporal ordering, multi-source data to reduce common method variance, and intervention studies to test whether psychological capital training combined with structured career development support can improve job embeddedness among early-career nurses.

## Data Availability

The raw data supporting the conclusions of this article will be made available by the authors, without undue reservation.
